# The inevitable inequality of cortical columns

**DOI:** 10.3389/fnsys.2022.921468

**Published:** 2022-09-20

**Authors:** Helen Barbas, Basilis Zikopoulos, Yohan J. John

**Affiliations:** ^1^Neural Systems Laboratory, Department of Health Sciences, Boston University, Boston, MA, United States; ^2^Department of Anatomy and Neurobiology, Boston University School of Medicine, Boston, MA, United States; ^3^Graduate Program in Neuroscience, Boston University and School of Medicine, Boston, MA, United States; ^4^Human Systems Neuroscience Laboratory, Department of Health Sciences, Boston University, Boston, MA, United States

**Keywords:** neuroanatomy, cortex, column, lamina, layer, primate, evolution

## Abstract

The idea of columns as an organizing cortical unit emerged from physiologic studies in the sensory systems. Connectional studies and molecular markers pointed to widespread presence of modular label that necessitated revision of the classical concept of columns. The general principle of cortical systematic variation in laminar structure is at the core of cortical organization. Systematic variation can be traced to the phylogenetically ancient limbic cortices, which have the simplest laminar structure, and continues through eulaminate cortices that show sequential elaboration of their six layers. Connections are governed by relational rules, whereby columns or modules with a vertical organization represent the feedforward mode of communication from earlier- to later processing cortices. Conversely, feedback connections are laminar-based and connect later- with earlier processing areas; both patterns are established in development. Based on studies in primates, the columnar/modular pattern of communication appears to be newer in evolution, while the broadly based laminar pattern represents an older system. The graded variation of cortices entails a rich variety of patterns of connections into modules, layers, and mixed arrangements as the laminar and modular patterns of communication intersect in the cortex. This framework suggests an ordered architecture poised to facilitate seamless recruitment of areas in behavior, in patterns that are affected in diseases of developmental origin.

## Introduction

The concept of cortical column emerged over 50 years ago from findings in the somatosensory system (reviewed in Mountcastle, 1997). In its initial description, the term captured Mountcastle’s observation that neurons recorded along vertical penetrations from the top cellular layer 2 to layer 6 responded to the same stimuli, such as light touch on the body surface (reviewed in Kaas, 2012). Mountcastle’s work was followed by findings from the primary visual cortex of cats and monkeys by Hubel and Wiesel (e.g., Hubel and Wiesel, 1968). The latter described vertical columns of neurons that preferentially respond to stimuli of a specific orientation. Columns of best frequency responses were also mapped on the primary auditory cortex of macaques (e.g., Merzenich and Brugge, 1973). The organization within the primary motor cortex was more complex, whereby evoked movement of a joint was clustered in “mini-columns,” while adjoining mini columns above or below evoked responses to a different joint, likely associated with the complex sequences required for responses in the cortical motor system (reviewed in Kaas, 2012).

The column concept thus began as a functional principle. The introduction of neural tracers to study connections revealed patterns that could be the anatomic correlates of physiologic columns, as seen widely throughout the cortex, including high-order association prefrontal areas (e.g., Bugbee and Goldman-Rakic, 1983; Rockland, 2010; Kaas, 2012; Casanova and Casanova, 2019). Connection studies and molecular markers sparked new debates as labeled patterns often were modular, resembling short columns. The cytochrome oxidase marker, for example, labeled patches (modules), found especially in layers 3 and 2 of primary visual cortex (V1), where neurons did not respond to a specific orientation of a visual stimulus, as neurons did above and below the blobs (Livingstone and Hubel, 1984). Debates about the features of columns or modules, such as extension of axons beyond their borders could be reconciled by the presence of inhibitory neurons, which can reduce extraneous responses on the flanks of active columns, attesting to their dynamic nature (da Costa and Martin, 2010; Rockland, 2010; Wang, 2020).

Neurons in columns or modules are strongly interconnected locally in the vertical direction, and are also innervated by fewer but highly consequential extrinsic connections from other cortices or from subcortical structures (Gilbert, 1983; Pandya et al., 1988; Callaway, 1998). Discussions about the organization of columns/modules have mostly proceeded outside such considerations, outside the context of the general principle of cortical systematic variation, and outside the intricate confluence of the vertical (columnar) and horizontal (laminar) organization of the cortex, which we address here.

### Columns vary as the cortex varies systematically

The general principle of cortical systematic variation is fundamental for explaining the inequality of cortical columns and modules. This principle was discovered by great thinkers working independently in different continents and with diverse species. Investigators that include Abbie (1939, 1940), Dart (1934), von Economo (1927/2009), and Sanides (1962, 1970, 1972) were able to see beyond the weeds of subtle differences in the local cytoarchitecture among cortical areas, to abstract the principle of systematic variation (for discussion of the ancestral dual allocortical areas, i.e., the olfactory cortex and the hippocampus, beyond which neocortical areas arise (see Pandya et al., 1988; Barbas, 2015; Garcia-Cabezas et al., 2019). In this scheme, differences across species reflect specializations. For example, in primates with frontally placed eyes central visual field emphasis allows detailed scene analysis and depth perception, reflected in the great laminar elaboration of V1. In rats and mice the primary visual cortex is by comparison rudimentary. Instead, the vibrissa somatosensory cortex shows specific elaboration, and is the only rodent area with a well-developed layer 4, affording these species a guide to sample the haptic environment and navigate in dark and narrow spaces. Nevertheless, systematic variation is a general principle, revealing systematic changes in laminar structure in each of the cortical systems, such as the visual, somatosensory, auditory, motor, and other cortices across species.

As an example, let us consider the ventral cortical visual system, where the changes in laminar organization are easiest to see because they follow an approximate posterior to anterior direction in primates. Accordingly, V1 has the best delineated six layers, with specialized subdivisions that reflect the precise mapping of the visual environment. The systematic cortical variation in laminar structure is seen along the entire series of visual association cortices (V2, V3, V4, and inferior temporal cortices), all of which are eulaminate but vary in laminar differentiation, often in neuronal density, and level of myelination (Pandya et al., 1988; Hilgetag et al., 2016). In the most anterior part of this axis, the rostral temporal pole does not have six layers: parts of it are dysgranular, which means that they have an ill-defined layer 4, and parts are agranular, meaning that they lack layer 4, and are poorly myelinated. Comparable gradual changes in laminar organization are seen in the dorsal cortical visual system, and in all other systems, such as the somatosensory, auditory, motor/premotor, and prefrontal cortical (PFC) systems. Interestingly, the latter two have ventral and dorsal specialized sectors as well (Barbas and Pandya, 1987, 1989).

There are thus parallels in the changes in cortical architecture in each of the cortical systems. As we piece together the gradual changes in laminar structure along the visual, auditory, somatosensory, and other cortices, we see that the entire cortex can be traced to a ring of primordial areas that unites the medial and basal surfaces and forms the base of the entire cortex. Adjacent to these phylogenetically ancient areas, eulaminate cortices with six layers emerge, and sequentially adjacent areas show gradual elaboration of their layers. Systematic variation in laminar structure can be seen in all systems, as is evident in primates, where the cortical expanse allows appreciation of changes.

The systematic variation in the ventral visual cortical system, is accompanied by physiological differences in the response properties of neurons. In V1 neurons have small receptive fields representing a small part of the visual periphery. Receptive fields gradually increase in size in areas from a posterior to anterior direction, as they map larger portions of the visual periphery (Gross, 1992). The contingencies for neuronal responses along the posterior to anterior axis gradually increase as well.

Complexity in visual and other high order association areas is conferred by more influences through projections from other structures, including the thalamus (e.g., Galuske et al., 2000; da Costa and Martin, 2010), which activate monosynaptically not only layer 4 but also the deep layers, at least in rats (Constantinople and Bruno, 2013). In primates, the most anterior inferior temporal visual areas combine a broad map of the visual field with visual memory (e.g., Gross, 1994). Moreover, the “canonical” influence of projections on the cortical column, described to innervate layer 4, then the supragranular layers and then the infragranular layers in visual cortex (reviewed in Douglas and Martin, 2004), shows a different sequence when the monkey is required to recall paired associations from memory (Miyashita, 2022).

More work needs to be done to understand the influences on the column from the thalamus in motor and the phylogenetically old limbic cortices. At a broad level, pathways from the thalamus emanate from more thalamic nuclei when they project to PFC areas that belong to the limbic ring than to lateral eulaminate PFC (Dermon and Barbas, 1994). The same pattern is seen for other subcortical structures, which have a broader reach on areas with simpler laminar architecture. For example, projections from the basal forebrain or the amygdala are comparatively sparser to lateral (eulaminate) PFC and are broader and denser to the PFC limbic ring in posterior orbital and anterior cingulate cortical (ACC) regions (Ghashghaei and Barbas, 2001); reviewed in Barbas (2015). It is likely that the extent of connections differs within the axis of differentiation within all cortical systems (Pandya et al., 1988). It is reasonable to expect that broader projections render processing more complex and increase the contingencies for response of neurons in areas within, or close to, the limbic ring.

### Feedforward connections are modular, feedback connections are laminar

Pathways in sensory systems have been grouped into “feedforward” and “feedback,” by analogy with the feedforward flow of signals from the sensory periphery to the thalamus, to primary areas, and then to sensory association cortices; feedback refers to connections in the opposite direction (reviewed in Felleman and Van Essen, 1991; Barbas, 2015). Studied most thoroughly in the visual cortical system, pathways from the thalamus innervate layer 4 of V1, and V1 innervates V2. Feedforward pathways are focal; they originate in the superficial layers close to layer 4 and innervate neurons in a short module in layer 4, which then projects focally to the middle layers in and around layer 4 in adjacent sensory association cortex, and so on. This account is oversimplified (see for example Siu et al., 2021).

Labeling with neural tracers revealed another feature of cortical connections, namely, that along with pathways proceeding in the feedforward direction, there are pathways going in the opposite direction, from later- to earlier processing areas. One such example is the projection from V2 to V1. Feedback connections have features that markedly differ from feedforward pathways: they originate in the deep layers and terminate as broad ribbons in layer 1 (Rockland and Pandya, 1979) as well as in adjacent layers 2 and upper layer 3, depending on the cortical areas involved (Barbas and Rempel-Clower, 1997).

The consistent patterns of connections seen in sensory and high-order association cortices made it possible to link their rich variety to the systematic variation of the cortex (Barbas, 1986; Barbas and Rempel-Clower, 1997); reviewed in Barbas (2015). What emerged from this analysis is a general rule that relates the laminar relationship of linked areas to the pattern of their interconnections, as summarized in the Structural Model. Accordingly, feedforward connections describe those that originate in an area with more complex laminar structure than the cortex of termination, and feedback refers to those that proceed in the opposite direction. Lateral connections occur between areas that have comparable laminar structure. Neighboring areas often have comparable laminar structure, but such relationships extend beyond neighborhood. Because systematic variation is seen in each cortical system, areas with comparable laminar structure occur across systems. Areas with comparable laminar structure are often connected, linking for example, PFC with temporal, parietal, or occipital areas, in patterns explained by relational rules based on architecture.

**FIGURE 1:**
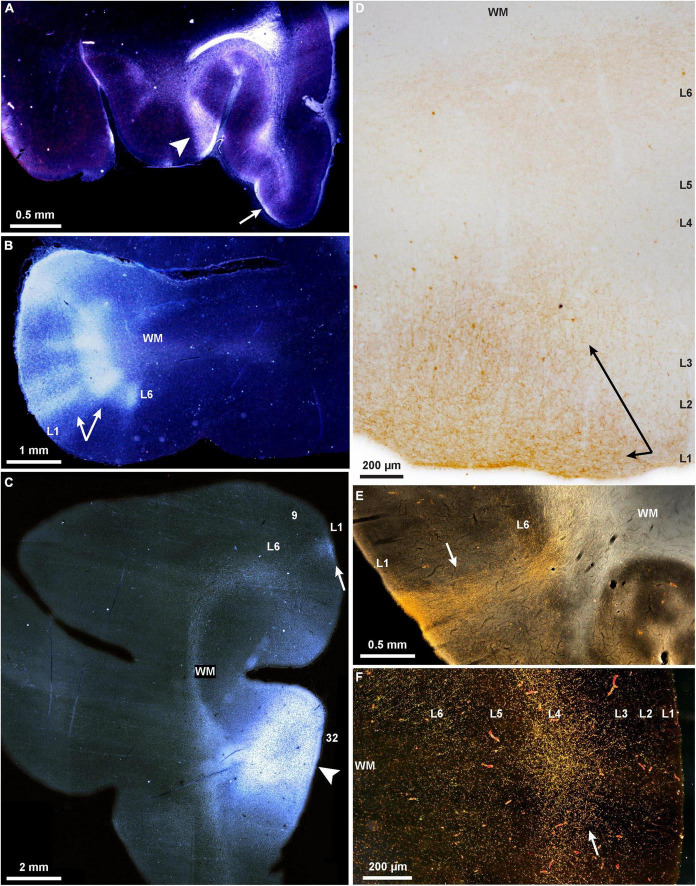
Projections show cortical columnar/modular, laminar, and complex mixed patterns in rhesus macaques. **(A–C)** Darkfield photomicrographs show bright labeled fibers traveling through the white matter to connect PFC areas; **(A)** A pathway from area 32 terminates in layer 1 of orbital areas 14 (arrow, feedback type), and in a complex column and layer 1 of area 13 (arrowhead, mixed lateral and feedback pattern); **(B)** A pathway from ventral area 46 terminates in area 12 in a complex columnar pattern with stronger label in the deep layers (arrows); **(C)** termination of the pathway from area 32 in area 9 is in layer 1 (arrow; feedback type); the injection of tritiated amino acids in area 32 is also seen here, arrowhead. **(D)** Brightfield photomicrograph shows golden brown labeled fibers and terminations in the upper layers of dorsolateral area 46 (arrows; broad feedback type that involves layer 1 and 2-3a) after injection of BDA tracer in area 32. **(E)** Darkfield photomicrograph shows bright labeled fibers traveling through the white matter and terminating in all layers of occipital visual area prostriata in a pathway from area 32 of medial PFC (arrow; complex columnar type with broader extent in layer 1; BDA label). **(F)** Darkfield photomicrograph shows bright labeled fibers and terminations of a pathway from the parvicellular part of the MD thalamic nucleus that terminates in the middle layers of lateral area 46 (arrow; feedforward type; tracer, HRP-WGA).

Some of the complex patterns of connections are shown in Figure 1. The example in Figure 1E shows a strong columnar termination in occipital area prostriata of a pathway that originates in medial PFC area 32. It illustrates that even though these two areas are situated far apart and in different lobes, their connections are strong, and of a columnar pattern, as predicted by their similar dysgranular structure, according to relational architectonic rules (reviewed in Barbas, 2015). In addition to pattern, the strength of connections can be predicted by the general relational rule: the strongest connections link areas of comparable structure, and their connections involve more layers (Barbas and Rempel-Clower, 1997). Areas that are dissimilar in structure are more sparsely connected and involve fewer layers. Thus, connections across the cortical expanse are governed by the same set of predictive rules in prefrontal, visual, parietal, temporal, motor, and other areas, notwithstanding their functional heterogeneity, or the local cellular architecture of the respective regions and areas.

In summary, the pattern of connections is linked to the general principle of cortical systematic variation throughout the cortex. The phylogenetically ancient areas of the limbic ring lie at the foot of each cortical system, and have broad influences on the evolving neocortex. The frequently named feedforward and feedback connections differ by influencing, respectively small columns/modules or broadly layers and in mixed patterns, which reveal the intersection and confluence of the two modes of communication.

## Discussion

The linkage of connections to the gradual changes in laminar structure in each of the cortical systems entails a large variety of patterns of connections as areas variously link with each other based on the relative difference/similarity in their laminar structure. The patterns of connections also reveal distinct types of communication. Feedforward connections are focal and the closest to a columnar pattern. On the other hand, feedback connections innervate broadly stretches of the cortex by layers. The principle of systematic cortical variation also helps explain why many connections show a mixed columnar and laminar pattern, and vary in the extrinsic influences that impinge on them. A more detailed exploration of connectivity models was beyond the scope of this brief perspective (but see, e.g., Markov et al., 2013; Beul et al., 2018); for a direct comparison of various models of connectivity (see Hilgetag et al., 2016).

### Columns and layers intersect in the cortex

The quasi-columnar feedforward terminations and the laminar-based feedback connections intersect in the cortex, creating mixed patterns reminiscent of the classical broad description that feedforward connections innervate layer 4, and feedback connections avoid layer 4 (reviewed in Felleman and Van Essen, 1991); for nuanced patterns based on the relative structural relationship of linked areas see (Hilgetag et al., 2016; Figures 1A,B). Complex patterns are also seen in the connections with the thalamus, which help refine areas through guide molecules in development (Price et al., 2006). Thalamic nuclei project focally to the middle cortical layers (Figure 1F), and broadly to layer 1 in all cortical systems. For example, layer 1 of V1 is innervated by projections from the thalamic lateral geniculate nucleus (LGN) that emanate from small cells in the koniocellular layers found between the six LGN layers (e.g., Hendry and Yoshioka, 1994; Hendry and Reid, 2000; Klein et al., 2016). In high-order association areas, such as PFC, terminations from the mediodorsal (MD) thalamic nucleus innervate strongly the middle layers as well as the upper layers (1-3a). The mixed pattern is accentuated in the projections from magnocellular (medial) MD, which projects to the posterior orbital and the ACC, the phylogenetically old prefrontal cortices. The latter receive projections from a larger variety of thalamic nuclei, which innervate the middle layers as well as the upper layers (Dermon and Barbas, 1994; McFarland and Haber, 2002; Xiao et al., 2009). Projections from the basal forebrain innervate widely but differentially the PFC, targeting mostly layer 1 of eulaminate lateral PFC, but robustly innervate all layers of the limbic ACC (Ghashghaei and Barbas, 2001).

The two systems of communication are also evident in the neurochemical features of thalamic projections. A core thalamic system originates in neurons labeled by the calcium binding protein parvalbumin (PV), which are excitatory in the thalamus (unlike the cortex), and project focally to the middle cortical layers (Jones, 1998). A matrix system is composed of neurons labeled by the calcium binding protein calbindin (CB), which project widely to the upper cortical layers, akin to feedback projections. Some thalamic nuclei are dominated by one of these two markers. Thalamic PV neurons predominate in nuclei that project to primary cortices. At the other extreme, some other thalamic nuclei, such as the midline, are dominated by CB neurons (e.g., Joyce et al., 2022). The midline (limbic) thalamic nuclei project widely to the upper cortical layers in PFC but also to other high-order association cortices. Many thalamic nuclei, such as the motor-related ventral anterior, have mixed and near equal distributions of PV and CB neurons (Zikopoulos and Barbas, 2007). Variability of columns or modules is thus inevitable in view of the systematic variation of the cortex. Cortical and thalamic pathways innervate to a variable extent cortical areas in mixed columnar and laminar arrays, thwarting attempts to rigidly categorize them by anatomy or function (e.g., Figures 1A,B).

The segregation into columns and layers is elusive, as much information is still lacking. For example, while the local projection of layer 4 within a column has been described across species, information is still lacking for the highly complex layer 5, which has projections within a column as well as with distant areas. It is unclear whether the same layer 5 neurons that project within a column also participate in the broadly described feedback corticocortical projections (e.g., Rockland and Pandya, 1979). In primates, the proportion of neurons that project to two areas is low, and most often seen in limbic areas (Barbas, 1995). The complexity of layer 5 neurons and their projection system awaits further study as is for other layers especially in primates.

### Developmental and evolutionary origin of two modes of communication

**FIGURE 2:**
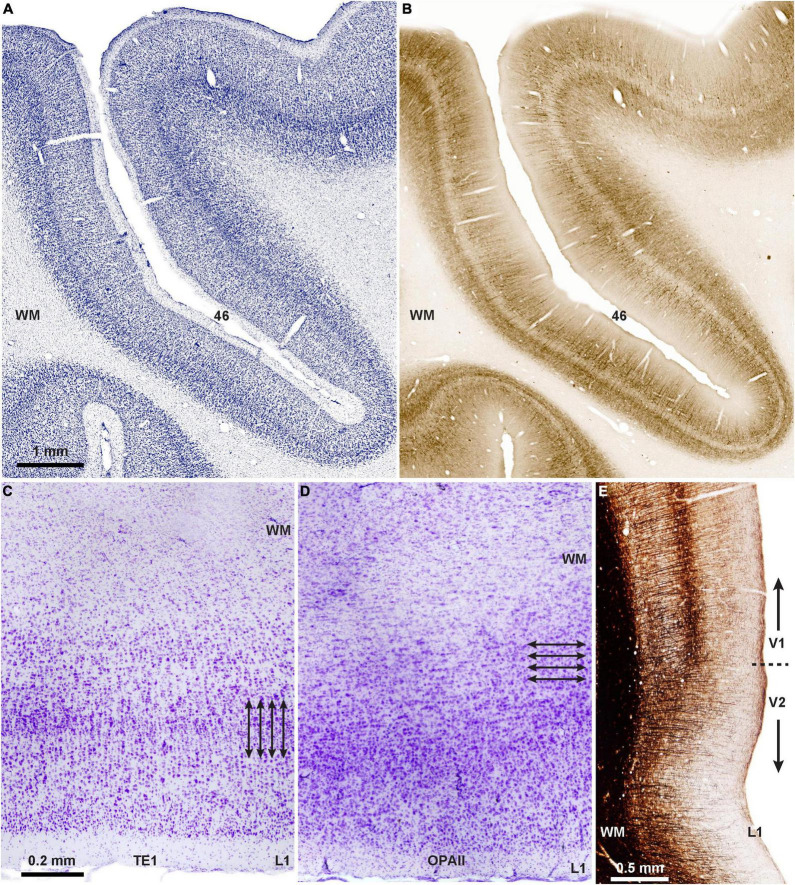
Two types of cortical architecture in macaque monkeys. **(A)** Columnar type of arrangement of neurons is seen in lateral PFC areas that have six layers (eulaminate), seen in Nissl-stained coronal section through PFC area 46 of the rhesus macaque; **(B)** adjacent matched coronal section immunostained for SMI-32; the direction of fibers in B suggests a columnar (modular) organization in this eulaminate area with a distinct layer 4 (unstained central ribbon in area 46). **(C)** Photomicrograph of Nissl-stained coronal section through inferior temporal area TE1 shows columnar organization of the cortex (arrows indicate the vertical arrangement of neurons into columns). **(D)** Photomicrograph of Nissl-stained coronal section through the agranular (limbic) orbital periallocortex (area OPAll) shows laminar organization of the cortex (arrows indicate the horizontal organization of neurons). **(E)** Myelin-stained coronal section shows the columnar direction of fibers in visual areas V1 and V2. Note the denser myelination in V1, which has the best delineated laminar organization in macaque and other primates. Myelin is an excellent marker for the graded differentiation of cortical areas: it is sparse in agranular and dysgranular areas and is enriched in eulaminate areas as they show graded changes in laminar structure.

The question arises as to the origin and functional significance of the modular and laminar systems of communication. The laminar pattern of communication may be phylogenetically older than the columnar, as suggested by the horizontal (laminar) orientation of neurons in limbic PFC area (area OPAll; Figure 2D), compared to the vertical (columnar/modular) arrangement of neurons in eulaminate areas (Figures 2A–C,E). Supporting evidence for this hypothesis is seen in the mostly feedback type connections of the ancient limbic cortices (Figure 1C) as they connect with eulaminate cortices (Joyce and Barbas, 2018; Joyce et al., 2020). Additional evidence is the predominance of matrix neurons in the limbic thalamic nuclei that project broadly to layer 1.

Further, in the visual system ocular dominance projections in the ancestral primate tree shrew are represented in layers, lacking the fine organization into blobs seen in the primary visual cortex of other primates (Horton and Adams, 2005; Kaas, 2012). Orientation selectivity appears in the primary visual cortex of all primates, including the tiny sized prosimian mouse lemur (Ho et al., 2021), as is the presence of modular blobs, suggesting early emergence in primate evolution (Saraf et al., 2019). Neurons that are selective for orientation are seen also in rodents, but are not organized into modules (reviewed in Ho et al., 2021).

How do the two systems of laminar and modular patterns of connections arise? The universal principle of systematic variation of cortical regions let us to suggest that they reflect differences in the timing of development. Accordingly, limbic cortices must complete their development first, which could help explain their overall simpler laminar structure (e.g., Dombrowski et al., 2001; Figure 2D). Limited developmental evidence in macaque monkeys (Rakic, 2002) is consistent with a sequential development of areas that vary along the spectrum of laminar differentiation. Genetic mechanisms must initiate cortical development, but as ontogenetically related neurons migrate to their place (Rubenstein and Rakic, 1999) and connect with other areas, stochastic processes may lead to self-organization of neurons into columns and modules seen in the adult cortex of primates and some other species.

Knowledge of the timing of development of areas is critical for theoretical and functional reasons. If areas develop and connect in an ordered sequence, it will provide a mechanism for the relational nature of the rules of the Structural Model (Barbas and Rempel-Clower, 1997). Most developmental studies have compared the progenitor zone between rodents and primates, noting expansion of the outer subventricular zone in primates, which gives rise to upper layer neurons (e.g., Geschwind and Rakic, 2013; Dehay et al., 2015). The systematic variation of the cortex suggests that the workings of evolution can be inferred in one brain. For example, recent evidence revealed that progenitor zones below prospective limbic cortices in the human embryo are thinner and less dense, and neurons differentiate earlier than in prospective eulaminate cortices (Barbas and García-Cabezas, 2016). Moreover, the classical findings that layer 1 is present at the onset of cortical development (Marin-Padilla, 1998) begins to explain the development of patterns of cortical connections. The earliest migrating neurons form layer 6, and as neurons reach to connect they innervate sites in layer 1, in a pattern akin to the feedback projections adopted by the limbic cortices. In this scenario, the phylogenetically ancient limbic areas influence the new in their laminar-based projections.

### Functional implications

Does the organization of functional units into columns and layers facilitate function? The intersection of layers and columns is entailed in the systematic variation of the cortex that governs the pattern of connections. Feedback connections unite older with newer areas in a relational pattern adopted by all cortices. This union may allow differential recruitment of neurons, ranging from focal driver input to the middle cortical layers from other cortices or the thalamus (e.g., Sherman, 2012), to nuanced activation of dendritic compartments in the upper layers (Gilman et al., 2017). The extensive cortical and subcortical feedback pathways that terminate in layer 1 meet the apical dendrites of local pyramidal neurons from the layers below, and may in time recruit adjacent areas in behavior (e.g., Zikopoulos and Barbas, 2007).

The functional significance of the intricate connection patterns is an on-going pursuit (e.g., Markov et al., 2013; Vezoli et al., 2021). More work is needed to understand the timing of development of areas that vary along the cortical spectrum, as well as the onset of their connections. From a health perspective, perturbation of the intricate process of neuronal migration that gives rise to the adult patterns has functional consequences. The unorthodox lamination in the reeler mouse is accompanied by marked disturbances in motor and other functions. Subtle or large differences in structure are seen also in diseases of developmental origin, including autism and schizophrenia. The exquisite patterns formed as layers and columns intersect, as in the warp and weft of a fine tapestry, may hold the secrets to their functional significance in health and disease.

## Data availability statement

The original contributions presented in this study are included in the article.

## Ethics statement

This is a perspective manuscript that reports findings from studies previously approved by the IACUC at Boston University and School of Medicine.

## Author contributions

HB: conceptualization and writing. BZ: conceptualization, figures, and manuscript review. YJ: conceptualization and manuscript review. All authors contributed to the article and approved the submitted version.

## References

[B1] Abbie A. A. (1939). The origin of the corpus callosum and the fate of the structures related to it. *J. Comp. Neurol.* 70 9–44. 10.1002/cne.900700103

[B2] Abbie A. A. (1940). Cortical lamination in the monotremata. *J. Comp. Neurol.* 72 429–467. 10.1002/cne.900720302

[B3] Barbas H. (1986). Pattern in the laminar origin of corticocortical connections. *J. Comp. Neurol.* 252 415–422. 10.1002/cne.902520310 3793985

[B4] Barbas H. (1995). Pattern in the cortical distribution of prefrontally directed neurons with divergent axons in the rhesus monkey. *Cereb. Cortex* 5 158–165. 10.1093/cercor/5.2.158 7620292

[B5] Barbas H. (2015). General cortical and special prefrontal connections: Principles from structure to function. *Annu. Rev. Neurosci.* 38 269–289. 10.1146/annurev-neuro-071714-033936 25897871

[B6] Barbas H. García-Cabezas M. A. (2016). How the prefrontal executive got its stripes. *Curr. Opin. Neurobiol.* 40 125–134. 27479655 10.1016/j.conb.2016.07.003 PMC5056826

[B7] Barbas H. Pandya D. N. (1987). Architecture and frontal cortical connections of the premotor cortex (area 6) in the rhesus monkey. *J. Comp. Neurol.* 256 211–218. 10.1002/cne.902560203 3558879

[B8] Barbas H. Pandya D. N. (1989). Architecture and intrinsic connections of the prefrontal cortex in the rhesus monkey. *J. Comp. Neurol.* 286 353–375. 10.1002/cne.902860306 2768563

[B9] Barbas H. Rempel-Clower N. (1997). Cortical structure predicts the pattern of corticocortical connections. *Cereb. Cortex* 7 635–646. 10.1093/cercor/7.7.635 9373019

[B10] Beul S. F. Goulas A. Hilgetag C. C. (2018). Comprehensive computational modelling of the development of mammalian cortical connectivity underlying an architectonic type principle. *PLoS Comput. Biol.* 14:e1006550. 10.1371/journal.pcbi.1006550 30475798 PMC6261046

[B11] Bugbee N. M. Goldman-Rakic P. S. (1983). Columnar organization of corticocortical projections in squirrel and rhesus monkeys: Similarity of column width in species differing in cortical volume. *J. Comp. Neurol.* 220 355–364. 10.1002/cne.902200309 6315786

[B12] Callaway E. M. (1998). Local circuits in primary visual cortex of the macaque monkey. *Annu. Rev. Neurosci.* 21 47–74. 10.1146/annurev.neuro.21.1.47 9530491

[B13] Casanova M. F. Casanova E. L. (2019). The modular organization of the cerebral cortex: Evolutionary significance and possible links to neurodevelopmental conditions. *J. Comp. Neurol.* 527 1720–1730. 10.1002/cne.24554 30303529 PMC6784310

[B14] Constantinople C. M. Bruno R. M. (2013). Deep cortical layers are activated directly by thalamus. *Science* 340 1591–1594. 10.1126/science.1236425 23812718 PMC4203320

[B15] da Costa N. M. Martin K. A. (2010). Whose cortical column would that be? *Front. Neuroanat.* 4:16. 10.3389/fnana.2010.00016 20640245 PMC2904586

[B16] Dart R. A. (1934). The dual structure of the neopallium: Its history and significance. *J. Anat.* 69 3–19. 17104513 PMC1249252

[B17] Dehay C. Kennedy H. Kosik K. S. (2015). The outer subventricular zone and primate-specific cortical complexification. *Neuron* 85 683–694. 25695268 10.1016/j.neuron.2014.12.060

[B18] Dermon C. R. Barbas H. (1994). Contralateral thalamic projections predominantly reach transitional cortices in the rhesus monkey. *J. Comp. Neurol.* 344 508–531. 10.1002/cne.903440403 7523458

[B19] Dombrowski S. M. Hilgetag C. C. Barbas H. (2001). Quantitative architecture distinguishes prefrontal cortical systems in the rhesus monkey. *Cereb. Cortex* 11 975–988. 10.1093/cercor/11.10.975 11549620

[B20] Douglas R. J. Martin K. A. (2004). Neuronal circuits of the neocortex. *Annu. Rev. Neurosci.* 27 419–451. 10.1146/annurev.neuro.27.070203.144152 15217339

[B21] Felleman D. J. Van Essen D. C. (1991). Distributed hierarchical processing in the primate cerebral cortex. *Cereb. Cortex* 1 1–47. 10.1093/cercor/1.1.1 1822724

[B22] Galuske R. A. Schlote W. Bratzke H. Singer W. (2000). Interhemispheric asymmetries of the modular structure in human temporal cortex. *Science* 289 1946–1949. 10.1126/science.289.5486.1946 10988077

[B23] Garcia-Cabezas M. A. Zikopoulos B. Barbas H. (2019). The Structural Model: A theory linking connections, plasticity, pathology, development and evolution of the cerebral cortex. *Brain Struct. Funct.* 224 985–1008. 10.1007/s00429-019-01841-9 30739157 PMC6500485

[B24] Geschwind D. H. Rakic P. (2013). Cortical evolution: Judge the brain by its cover. *Neuron* 80 633–647. 10.1016/j.neuron.2013.10.045 24183016 PMC3922239

[B25] Ghashghaei H. T. Barbas H. (2001). Neural interaction between the basal forebrain and functionally distinct prefrontal cortices in the rhesus monkey. *Neuroscience* 103 593–614. 10.1016/S0306-4522(00)00585-6 11274781

[B26] Gilbert C. D. (1983). Microcircuitry of the visual cortex. *Annu. Rev. Neurosci.* 6 217–247. 10.1146/annurev.ne.06.030183.001245 6132585

[B27] Gilman J. P. Medalla M. Luebke J. I. (2017). Area-specific features of pyramidal neurons-a comparative study in mouse and Rhesus Monkey. *Cereb. Cortex* 27 2078–2094. 10.1093/cercor/bhw062 26965903 PMC6059164

[B28] Gross C. G. (1992). Representation of visual stimuli in inferior temporal cortex. *Philos. Trans. R. Soc. B* 335 3–10. 10.1098/rstb.1992.0001 1348134

[B29] Gross C. G. (1994). How inferior temporal cortex became a visual area. *Cereb. Cortex* 5 455–469. 10.1093/cercor/4.5.455 7833649

[B30] Hendry S. H. Reid R. C. (2000). The koniocellular pathway in primate vision. *Annu. Rev. Neurosci.* 23 127–153. 10.1146/annurev.neuro.23.1.127 10845061

[B31] Hendry S. H. Yoshioka T. (1994). A neurochemically distinct third channel in the macaque dorsal lateral geniculate nucleus. *Science* 264 575–577. 10.1126/science.8160015 8160015

[B32] Hilgetag C. C. Medalla M. Beul S. Barbas H. (2016). The primate connectome in context: Principles of connections of the cortical visual system. *NeuroImage* 134 685–702. 10.1016/j.neuroimage.2016.04.017 27083526 PMC5135480

[B33] Ho C. L. A. Zimmermann R. Florez Weidinger J. D. Prsa M. Schottdorf M. Merlin S. (2021). Orientation preference maps in *Microcebus murinus* reveal size-invariant design principles in primate visual cortex. *Curr. Biol.* 31 733–741.e7. 10.1016/j.cub.2020.11.027 33275889 PMC9026768

[B34] Horton J. C. Adams D. L. (2005). The cortical column: A structure without a function. *Philos Trans R Soc Lond B Biol Sci* 360 837–862. 10.1098/rstb.2005.1623 15937015 PMC1569491

[B35] Hubel D. H. Wiesel T. N. (1968). Receptive fields and functional architecture of monkey striate cortex. *J.Physiol.* 195 215–243. 10.1113/jphysiol.1968.sp008455 4966457 PMC1557912

[B36] Jones E. G. (1998). Viewpoint: The core and matrix of thalamic organization. *Neuroscience* 85 331–345. 10.1016/S0306-4522(97)00581-2 9622234

[B37] Joyce M. K. P. Garcia-Cabezas M. A. John Y. J. Barbas H. (2020). Serial Prefrontal Pathways Are Positioned to Balance Cognition and Emotion in Primates. *J. Neurosci.* 40 8306–8328. 10.1523/JNEUROSCI.0860-20.2020 32989097 PMC7577604

[B38] Joyce M. K. P. Marshall L. G. Banik S. L. Wang J. Xiao D. Bunce J. G. (2022). Pathways for memory, cognition and emotional context: Hippocampal, subgenual Area 25, and Amygdalar Axons Show Unique Interactions in the Primate Thalamic Reuniens Nucleus. *J. Neurosci.* 42 1068–1089. 10.1523/JNEUROSCI.1724-21.2021 34903572 PMC8824507

[B39] Joyce M. P. Barbas H. (2018). Cortical connections position primate area 25 as a keystone for interoception, emotion, and memory. *J. Neurosci.* 38 1677–1698. 10.1523/JNEUROSCI.2363-17.2017 29358365 PMC5815452

[B40] Kaas J. H. (2012). Evolution of columns, modules, and domains in the neocortex of primates. *Proc. Natl. Acad. Sci. U.S.A.* 109(Suppl. 1), 10655–10660. 10.1073/pnas.1201892109 22723351 PMC3386869

[B41] Klein C. Evrard H. C. Shapcott K. A. Haverkamp S. Logothetis N. K. Schmid M. C. (2016). Cell-targeted optogenetics and electrical microstimulation reveal the primate koniocellular projection to supra-granular visual cortex. *Neuron* 90 143–151. 10.1016/j.neuron.2016.02.036 27021172

[B42] Livingstone M. S. Hubel D. H. (1984). Anatomy and physiology of a color system in the primate visual cortex. *J. Neurosci.* 4 309–356. 10.1523/JNEUROSCI.04-01-00309.1984 6198495 PMC6564760

[B43] Marin-Padilla M. (1998). Cajal-Retzius cells and the development of the neocortex. *Trends Neurosci.* 21 64–71. 10.1016/S0166-2236(97)01164-8 9498301

[B44] Markov N. T. Ercsey-Ravasz M. Van Essen D. C. Knoblauch K. Toroczkai Z. Kennedy H. (2013). Cortical high-density counterstream architectures. *Science* 342:1238406. 10.1126/science.1238406 24179228 PMC3905047

[B45] McFarland N. R. Haber S. N. (2002). Thalamic relay nuclei of the basal ganglia form both reciprocal and nonreciprocal cortical connections, linking multiple frontal cortical areas. *J. Neurosci.* 22 8117–8132. 10.1523/JNEUROSCI.22-18-08117.2002 12223566 PMC6758100

[B46] Merzenich M. M. Brugge J. F. (1973). Representation of the cochlear partition on the superior temporal plane of the macaque monkey. *Brain Res.* 50 275–296. 10.1016/0006-8993(73)90731-2 4196192

[B47] Miyashita Y. (2022). Operating principles of the cerebral cortex as a six-layered network in primates: Beyond the classic canonical circuit model. *Proc. Jpn. Acad. Ser. B Phys. Biol. Sci.* 98 93–111. 10.2183/pjab.98.007 35283409 PMC8948418

[B48] Mountcastle V. B. (1997). The columnar organization of the neocortex. *Brain* 120(Pt 4), 701–722. 10.1093/brain/120.4.701 9153131

[B49] Pandya D. N. Seltzer B. Barbas H. (1988). “Input-output organization of the primate cerebral cortex,” in *Comparative Primate Biology, Vol. 4: Neurosciences*, eds Steklis H. D. Erwin J. (New York, NY: Alan R. Liss), 39–80.

[B50] Price D. J. Kennedy H. Dehay C. Zhou L. Mercier M. Jossin Y. (2006). The development of cortical connections. *Eur. J. Neurosci.* 23 910–920. 10.1111/j.1460-9568.2006.04620.x 16519656

[B51] Rakic P. (2002). Neurogenesis in adult primate neocortex: An evaluation of the evidence. *Nat. Rev. Neurosci.* 3 65–71. 10.1038/nrn700 11823806

[B52] Rockland K. S. (2010). Five points on columns. *Front. Neuroanat.* 4:22. 10.3389/fnana.2010.00022 20589097 PMC2893004

[B53] Rockland K. S. Pandya D. N. (1979). Laminar origins and terminations of cortical connections of the occipital lobe in the rhesus monkey. *Brain Res.* 179 3–20. 10.1016/0006-8993(79)90485-2 116716

[B54] Rubenstein J. L. Rakic P. (1999). Genetic control of cortical development. *Cereb. Cortex* 9 521–523. 10.1093/cercor/9.6.521 10498269

[B55] Sanides F. (1962). [Architectonics of the human frontal lobe of the brain. With a demonstration of the principles of its formation as a reflection of phylogenetic differentiation of the cerebral cortex]. *Monogr. Gesamtgeb. Neurol. Psychiatr.* 98 1–201. 13976313

[B56] Sanides F. (1970). “Functional architecture of motor and sensory cortices in primates in the light of a new concept of neocortex evolution,” in *The primate brain: Advances in primatology*, eds Noback C. R. Montagna W. (New York, NY: Appleton-Century-Crofts Educational Division/Meredith Corporation), 137–208.

[B57] Sanides F. (1972). “Representation in the cerebral cortex and its areal lamination pattern,” in *The structure and function of nervous tissue*, ed. Bourne G. H. (New York, NY: Academic Press), 329–453. 10.1016/B978-0-12-119285-3.50013-1

[B58] Saraf M. P. Balaram P. Pifferi F. Gamanut R. Kennedy H. Kaas J. H. (2019). Architectonic features and relative locations of primary sensory and related areas of neocortex in mouse lemurs. *J. Comp. Neurol.* 527 625–639. 10.1002/cne.24419 29484648 PMC6109619

[B59] Sherman S. M. (2012). Thalamocortical interactions. *Curr. Opin. Neurobiol.* 22 575–579. 10.1016/j.conb.2012.03.005 22498715 PMC3398163

[B60] Siu C. Balsor J. Merlin S. Federer F. Angelucci A. (2021). A direct interareal feedback-to-feedforward circuit in primate visual cortex. *Nat. Commun.* 12:4911. 10.1038/s41467-021-24928-6 34389710 PMC8363744

[B61] Vezoli J. Magrou L. Goebel R. Wang X. J. Knoblauch K. Vinck M. (2021). Cortical hierarchy, dual counterstream architecture and the importance of top-down generative networks. *Neuroimage* 225:117479. 10.1016/j.neuroimage.2020.117479 33099005 PMC8244994

[B62] von Economo C. (1927/2009). *Cellular structure of the human cerebral cortex*, ed. Triarhou L. C trans. (Basel: Karger).

[B63] Wang X. J. (2020). Macroscopic gradients of synaptic excitation and inhibition in the neocortex. *Nat Rev Neurosci* 21 169–178. 10.1038/s41583-020-0262-x 32029928 PMC7334830

[B64] Xiao D. Zikopoulos B. Barbas H. (2009). Laminar and modular organization of prefrontal projections to multiple thalamic nuclei. *Neuroscience* 161 1067–1081. 10.1016/j.neuroscience.2009.04.034 19376204 PMC2700123

[B65] Zikopoulos B. Barbas H. (2007). Parallel driving and modulatory pathways link the prefrontal cortex and thalamus. *PLoS One* 2:e848. 10.1371/journal.pone.0000848 17786219 PMC1952177

